# Oral Health-Related Quality of Life in Adult Patients with Depression or Attention Deficit Hyperactivity Disorder (ADHD)

**DOI:** 10.3390/jcm12227192

**Published:** 2023-11-20

**Authors:** Stephan Gemp, Dirk Ziebolz, Rainer Haak, Nicole Mauche, Madlen Prase, Ezgi Dogan-Sander, Frauke Görges, Maria Strauß, Gerhard Schmalz

**Affiliations:** 1 Department of Cariology, Endodontology and Periodontology, University of Leipzig, 04103 Leipzig, Germany; stephan.gemp@medizin.uni-leipzig.de (S.G.); rainer.haak@medizin.uni-leipzig.de (R.H.); 2 Department of Psychiatry and Psychotherapy, University of Leipzig, 04103 Leipzig, Germany; nicole.mauche@medizin.uni-leipzig.de (N.M.); madlen.prase@medizin.uni-leipzig.de (M.P.); ezgi.dogan@medizin.uni-leipzig.de (E.D.-S.); frauke.goerges@medizin.uni-leipzig.de (F.G.); maria.strauss@medizin.uni-leipzig.de (M.S.)

**Keywords:** affective disorders, oral health-related quality of life, psychosocial impact, oral health impact profile

## Abstract

The aim of this cross-sectional study was the evaluation of the oral health-related quality of life (OHRQoL) in patients with depression or attention-deficit/hyperactivity disorder (ADHD) in comparison with a group of mentally healthy individuals. Patients from the Department of Psychiatry and Psychotherapy, University of Leipzig, Germany, were recruited. A healthy comparison group (HC) was recruited from the Department of Cariology, Endodontology and Periodontology. The OHRQoL was assessed using the Oral Health Impact Profile G14 (OHIP G14). Furthermore, a questionnaire regarding oral hygiene behaviour was applied. A total of 141 patients with depression or ADHD (depression n = 94, ADHD n = 47) and 145 HC individuals with a balanced age and gender distribution were surveyed. OHIP G14 median scores were significantly higher in the overall psychiatric patient group compared to HC (5.00 vs. 0.00, *p* < 0.001). This was also found for the four dimensions of OHIP G14 (*p* < 0.001). The OHIP G14 sum score of patients with depression and ADHD was comparable (5.00 vs. 6.50, *p* = 0.302). A significant association among psychiatric patients between smoking, gum bleeding, professional tooth cleaning, oral health education, interdental cleaning, and elevated OHIP scores was found (*p* < 0.001). In conclusion, patients with depression and adults with ADHD show a reduced OHRQoL. A contradictory association between oral hygiene/oral health behaviour and OHRQoL supports the hypothesis of a changed perception of oral conditions in patients with mental diseases. Interdisciplinary collaboration between psychiatric specialists and dentists should be fostered.

## 1. Introduction

Oral and mental health are in a potential bidirectional relationship. It has already been suggested that mental disorders affect the occurrence of oral diseases and vice versa [1]. Thereby, behavioural changes (reduced oral hygiene), smoking and alcohol consumption, stress, and potentially microbiological changes are factors that negatively affect oral conditions, especially periodontal diseases in patients with different mental disorders [2]. Moreover, a systematic review concluded that increased attention would be needed to support the oral health of those patients [3].

Oral health is not limited to physical issues. The patient’s perception of his or her oral conditions, reflected by the oral health-related quality of life (OHRQoL), has become of clinical importance, too [4]. Thereby, the OHRQoL can be influenced not only by physical oral diseases but also by psychosocial factors [5,6], making this issue interesting for patients with psychiatric disorders. In a representative adult German sample, associations between reduced OHRQoL and depression and/or anxiety were confirmed [7]. This underlines a potential relationship between OHRQoL and depressive disorders. Moreover, it has already been shown that outpatients (i.e., patients attending ambulant care/therapy for their psychiatric disease) with psychiatric disorders would show a diagnosis-dependent impairment of their OHRQoL [8]. With regard to affective disorders, a recent longitudinal study revealed that the exacerbation of depression was related to worse OHRQoL [9]. Another cross-sectional study found that depression, anxiety, and stress were associated with both periodontal disease severity and OHRQoL [10]. This appears particularly relevant for elderly individuals [11]. Altogether, an association between OHRQoL and depressive disorders appears plausible. Moreover, this would be of clinical relevance, as it might influence oral care. Therefore, an understanding of the patient’s perspective would be helpful to develop more patient-oriented oral care concepts. In this context, there is still limited knowledge in this field. Studies on depressive disorders often focus on the elderly [11,12]; patients with specific conditions, like drug addiction [13]; or students [14]. Systematically assessed data on in- and outpatients with severe depressive disorders are still rare.

Another patient group with an affective disorder might be of clinical importance in the dental context, i.e., adults with attention-deficit/hyperactivity disorder (ADHD). ADHD is a neurodevelopmental disorder with a remarkable prevalence of 5% in children and adolescents. Symptoms include different presentations of inattention and hyperactivity/impulsivity [15]. It has been reported that children with ADHD show a high incidence of caries and dental injuries [16]. Considering the effect of caries on OHRQoL in children [17], this issue could be particularly relevant in ADHD patients. Only one study on children with ADHD considered the OHRQoL as one outcome parameter, showing a reduced OHRQoL in this group [18]. ADHD does not only affect children but also adults as well [19]. Until now, there has been no study that has examined OHRQoL in adults with ADHD. Thus, there appears to be a research gap regarding oral health issues in this particular cohort, leading to a need for studies in this field.

Taken together, this current study aimed at the evaluation of OHRQoL in a cohort of in- and outpatients with depression or ADHD. Thereby, the results were compared between patients with depression and/or ADHD and, furthermore, with a healthy comparison group. Additionally, selected parameters regarding oral health behaviours should be considered within the group of patients with mental disorders to reveal potential influential factors on OHRQoL in those individuals. Thereby, the primary hypothesis was that adult patients with depression or ADHD would show a reduced OHRQoL compared to healthy patients. As a secondary hypothesis, it was assumed that the OHRQoL of mentally diseased patients would be influenced by the oral hygiene and dental behaviour of the patients.

## 2. Methods

### 2.1. Study Design

This current study was designed as a cross-sectional study, whereby a descriptive part (OHRQoL of patients with depression or ADHD) and an analytic part (associations between oral health behaviour and OHRQoL in the cohorts) were executed. The study protocol followed the ‘Strengthening the Reporting of Observational Studies in Epidemiology (STROBE)’ guidelines for reporting observational studies [20]. All patients were informed verbally and in written form about the study and gave a full informed consent for participation in the current examination. The study protocol was reviewed and approved by the ethics committee of the medical faculty of the University of Leipzig, Germany (Application No. 018/22-ek). The study was performed in accordance with the Declaration of Helsinki.

### 2.2. Study Participants

All patients who suffered from depression and/or ADHD attending the Department of Psychiatry of the University Medical Center in Leipzig between June 2022 and May 2023 were asked for their voluntary participation during their in- or outpatient stay. The patients were asked to complete different questionnaires after the treating specialist had assessed them as stable enough to cope with the additional participation in the study. It was aimed to include as many patients as possible; a power calculation was not applied. Inclusion criterion was a confirmed diagnosis of a major depression or ADHD according to ICD-10, as well as inpatient (in recent stationary care/therapy) or outpatient treatment (ambulant care) by the Ward for Affective Disorders at the Center for Psychiatry of the University Medical Center. The diagnosis was confirmed by the ward’s psychiatrist and psychologist. Only dentate individuals were included. In addition, no patients could be considered who had extensive dental prosthetic pre-treatment. Further in- and exclusion criteria were not specified.

For comparison, a respective group of persons without known or diagnosed mental disease was selected from the patient collective of the Department of Cariology, Endodontology and Periodontology of the University Medical Center in Leipzig. These patients were recruited consecutively, without any randomization or matching process. Thereby, mental health was evaluated within the medical history of the patients, which was also used to exclude potential confounders. It was aimed to recruit a group with a similar age and gender distribution as in the disease groups. The patients were surveyed during their routine appointments in the dental clinic. Further criteria for participation were equal for the patients with psychiatric disorders.

Age and gender were recorded as demographic data. Furthermore, data on previous and current medical or dental treatments, medication, oral health-related behaviour and perception were collected. The information was supplemented by a periodontitis questionnaire and a questionnaire to assess OHRQoL.

### 2.3. Oral Health Impact Profile (OHIP G14)

To assess OHRQoL, all participants answered a standardized and validated questionnaire. Thus, the German abbreviated form of the Oral Health Impact Profile (OHIP G14) was used to assess OHRQoL [21,22,23]. The OHIP G14 includes a report on the occurrence of 14 functional and psychosocial impairments that patients had experienced in the past month with respect to discomfort with their teeth, mouth, or dentures. A score on a five-point scale was provided for each question, whereby the following options were included: very often = ‘4’, fairly often = ‘3’, occasionally = ‘2’, hardly ever = ‘1’, and never = ‘0’. The maximum score of the OHIP G14 ranged from ‘0’ (all questions were answered ‘never’) to ‘56’ (all questions were answered ‘very often’). A mean score of two or more points in the sum score was interpreted as clinically relevant, according to the principle of minimally important difference (MID) [24]. Alongside the sum score, the four different dimensions, ‘oral function’, ‘psychosocial impact’, ‘oral pain’, and ‘orofacial appearance’ of the OHIP 14 [5], were considered.

### 2.4. Periodontitis and Oral Health Behaviour

**Figure 1:**
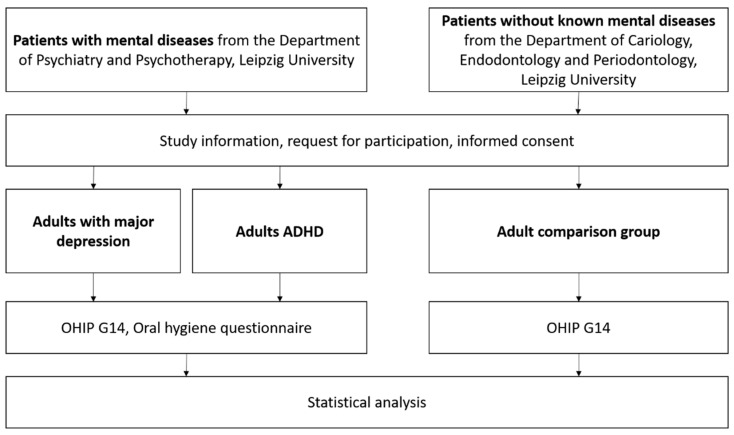
Study flow of the current study.

To assess periodontal complaints, oral health behaviour, and oral hygiene habits, a questionnaire was composed. This questionnaire was designed based on previous studies of this working group [25]. The questionnaire included questions regarding gingival bleeding, worsened taste, previous periodontal therapy, regular dental visits, and tooth cleaning, as well as whether the dentist was informed on the psychiatric disease and if patients were informed of the potential relationship between oral health and psychiatric diseases. Moreover, questions on oral hygiene behaviour were included, whereby interdental cleaning should serve as a marker for well-performed oral hygiene. An English version of the questionnaire is attached as Supplementary Materials. The elaborated oral hygiene questionnaire was only applied to patients with psychiatric diseases. The study flow is given in Figure 1.

### 2.5. Statistical Analysis

Statistical analysis was performed using SPSS, Version 29.0 (IBM, New York, NY, USA). The normal distribution of the data was tested using Kolmogorov–Smirnov test. Metric variables are presented as the mean values and standard deviations; categorical data are presented as percentage. Central tendencies of rank scaled variables are represented by median. For comparison of the groups with respect to their membership in a study group and OHIP-G14 scores, a Mann–Whitney U test and Kruskal–Wallis test were used, respectively. To analyse the oral health behaviour and sensation, the percentage of affirmed answers was considered. The relation between elevated OHIP G14 sum scores and oral health behaviour and sensation was observed using Wilcoxon test. The significance level was set at *p* < 0.05.

## 3. Results

### 3.1. Patients

In this current study, out of 184 patients, 141 individuals (participation rate: 76.6%) who had received psychiatric treatment as inpatients or outpatients were surveyed. A larger group (n = 94) had previously been diagnosed with a depressive disorder, and a smaller group (n = 47) had been diagnosed with ADHD. Within the two psychiatric subgroups, the proportion of women varied between 33 and 44.7%, with the overall sample having a female proportion of 36.9%. The mean age was 39 ± 13.1 in the psychiatric sample and varied between 36 and 40.9 years in the subgroups, with subjects ranging from 21 to 67 years of age. The healthy comparison group included 145 individuals with a mean age of 38.9 ± 13.9 years with a range of 21–67 years, which was similar to the psychiatric group. The proportion of women was 62.1%. A statistical analysis of age and gender did not reveal any significant differences between the two study groups (Table 1).

### 3.2. OHIP G14

**Figure 2:**
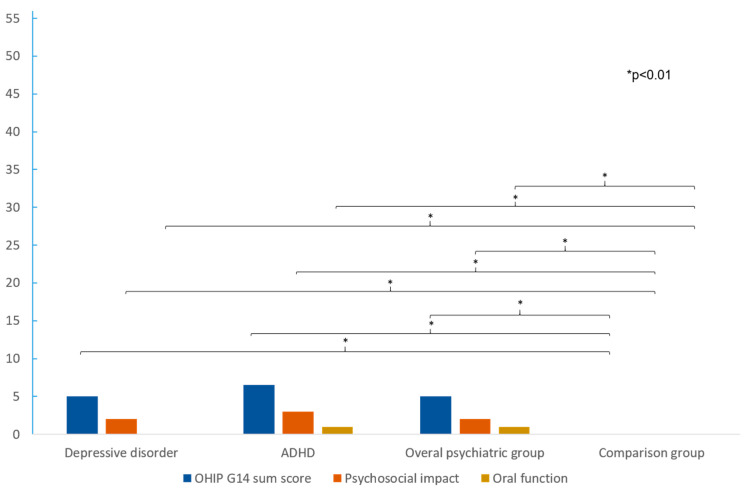
Comparison of the median values between the different groups. It can be seen that patients with mental disease showed significantly worse OHRQoL, without differences between ADHD and depressive disorder.

The resulting median scores in the OHIP G14 questionnaire were found to be significantly higher in the overall psychiatric group compared to the healthy comparison group (5.00 vs. 0.00, *p* < 0.001; Table 2, Figure 2). Median deviations were found in the sum of the items of oral function as well as in the sum of the items of psychosocial impairment (*p* < 0.001). Consideration of the two psychiatric subgroups revealed a larger deviation from the comparison group within the ADHD group in the total score (6.50 vs. 0, *p* < 0.001; Table 2, Figure 2). The median difference was more pronounced in psychosocial impact than in oral function (1.00 vs. 0; 3.00 vs. 0, *p* < 0.001; Table 2, Figure 2). A comparison of the psychiatric subgroups with each other showed no significant median differences, except for the OHIP Item ‘Taste worsened’ (*p* = 0.034; Table 2).

### 3.3. Oral Health Behaviour and Sensation

An analysis of the questions concerning oral health behaviour and oral sensation within the psychiatric sample revealed that a worse taste was more often affirmed within the ADHD group, which was already evident in the OHIP analysis (55.9% vs. 69.6%; Table 3). Similarly, the patients in the ADHD subgroup reported fewer regular dental visits (27.7% vs. 57.6%, Table 3) and fewer professional teeth cleanings (36.2% vs. 51.6%, Table 3) than the depressive disorders group. In addition, patients suffering from depressive disorders reported feeling better educated about oral hygiene and were more likely to practice interdental hygiene and mouth rinsing (Table 3).

### 3.4. Associations between OHIP and Oral Health Behaviour and Sensation

Further analysis of the association of elevated OHIP scores and oral health behaviours and sensations indicated a significant association among psychiatric patients between smoking, the incidence of gum bleeding, the use of professional dental cleaning, oral health education, interdental space cleaning measures, and elevated OHIP scores (*p* < 0.001; Table 4).

## 4. Discussion

### 4.1. Summary of the Main Results

This current cross-sectional study revealed a clinically relevant (difference in median of more than two in OHIP G14 sum score) and statistically significant difference in the OHRQoL of patients with depressive disorders and ADHD compared with healthy individuals. Thereby, patients with depressive disorders did not differ from adults with ADHD. As depicted in Table 2, the psychosocial dimension appears especially affected in the overall patient group. Oral health-related issues were associated with OHIP G14 in the cohort of patients with psychiatric diseases. While oral complaints (gum bleeding) were associated with good OHRQoL, regular professional tooth cleaning, education for oral health issues with regard to psychiatric diseases, and interdental cleaning were associated with worse OHRQoL.

### 4.2. OHRQoL Disparities in Patients with Depressive Disorders and ADHD

The results of the current study show certain conspicuous issues in the cohort of patients with depression or ADHD. First, the worse OHRQoL of these patients needs discussion. The available literature on patients with affective disorders supports the results of the current study, as they show a reduced OHRQoL in those individuals [7,8,9,10,11]. A study on the Japanese elderly showed that depressive symptoms were associated with a more than five-fold higher risk of impaired OHRQoL [12]. Another longitudinal study showed that an exacerbation of depression would be negatively associated with OHRQoL in the elderly [9]. However, the available literature consists of heterogeneous samples, whereby specific cohorts like elderly individuals or substance users were often included. This limits the comparability with the current sample. Nevertheless, the worse OHRQoL in psychiatric patients appears plausible and substantiated by the literature. For the first time, this current study explicitly examined adult patients with ADHD regarding their OHRQoL. In this cohort, the OHRQoL was particularly worse. Comparable literature for adults with ADHD is missing. Only one study in children with ADHD showed that their OHRQoL was insufficient [18]. Overall, the first hypothesis of this study, i.e., the reduced OHRQoL of patients with depression or ADHD, was confirmed by the abovementioned findings.

### 4.3. Factors Influencing OHRQoL and Mentally Diseased Patients

It is well known that oral diseases, including periodontitis, tooth loss, or temporomandibular diseases, negatively affect OHRQoL in general population samples [26,27,28,29]. Moreover, it is documented that those oral diseases are common in patients with psychiatric diseases [1,2,3]. While no statistically significant differences were confirmed between patients with depression and participants with ADHD, similar problems regarding oral health appear of importance for both groups. Thereby, Goh et al. (2022) showed that depression, anxiety, and stress had a negative impact on periodontitis and OHRQoL [10]. This supports the relationship between poor oral health and worse OHRQoL in patients with psychiatric diseases. In children with ADHD, OHRQoL, as well as oral health, were insufficient [18]. Furthermore, a recent meta-analysis summarized poor oral health, noting a high prevalence of dental caries in children with ADHD [30]. Until now, there is no comparable literature for the OHRQoL of adults with ADHD; however, the physical oral health of those patients has been evaluated in some studies. Thereby, it was concluded that adults with ADHD show poor oral health, which is potentially related to their impulsivity (impairment of oral hygiene), cigarette addiction, and sugar consumption [31]. Moreover, an increased prevalence of dental anxiety was found in adults with ADHD [32]. This appears in line with the current study´s findings, whereby less than 30% of participants with ADHD reported regularly visiting their dentist (see Table 3). Therefore, the insufficient oral condition could explain the reduced OHRQoL in those individuals.

On the other hand, a response shift might also be conceivable for this particular patient group. The concept of a response shift originates from Sprangers and Schwartz (1999) and describes a change in the patient’s individual perception [33]. Thereby, patients perceive health changes with regard to an internal standard, which is influenced by internal (e.g., chronic diseases) or external (e.g., environmental factors) parameters [34]. In the dental context, this would indicate that patients with chronic general disease would perceive their oral health condition differently from healthy individuals because the underlying general disease affects the internal standard of perception. This has already been described in dentistry for the first time in patients after solid organ transplantation [35]. This could be similar for patients with psychiatric diseases, potentially explaining the current study’s findings. The pronounced affection of psychosocial impact as one dimension of OHRQoL in both affective disorders and ADHD appears interesting and supportive in this context. It does not appear surprising that patients with a psychiatric disease show an affected psychosocial dimension of OHRQoL. Although the comparability is limited, the psychosocial impact has already been shown to be influenced by other chronic diseases. For example, patients under chronic hemodialysis show a particularly affected psychosocial impact of the OHRQoL, especially at the beginning of their dialysis [36]. Similarly, different groups of patients with rheumatic diseases [37], chronic kidney failure [38], or solid organ transplantation [35] show affected OHRQoL, especially in the psychosocial impact dimension. Further, the majority of studies reported that this was independent of their oral status. Therefore, previously, it has been concluded that patients with chronic diseases show a response shift regarding their oral health perception, whereby the general and psychosocial burden of their systemic chronic disease affects the OHRQoL, while patients develop a neglect of oral conditions. As mentioned above, this could be similar in patients with psychiatric diseases.

A further argument for this hypothesis is the finding that regular professional tooth cleaning, education for oral health issues with regard to psychiatric diseases, and interdental cleaning were associated with worse OHRQoL. Those parameters would argue for increased attention to oral health issues. Thereby, the secondary hypothesis regarding the association of oral hygiene and/or oral health behaviour with OHRQoL of mentally diseased patients can be seen as confirmed. However, this association was contradictory, leading to a perceived mismatch between patients’ perceptions and clinical situation. Potentially, patients with good oral health behaviour might recognize their oral problems, while the other group with reduced oral health behaviour might suppress oral health complaints, resulting in a good OHRQoL. Thus, the OHRQoL of patients with psychiatric diseases could be multifactorially influenced by their insufficient oral health, their psychosocial burden, and potentially by a kind of response shift regarding their perception of oral diseases and respective treatment needs. In the absence of an oral examination and further evaluation of those issues, this remains speculative.

### 4.4. Implications for Dental Care

Taken together, in clinical consequence, patients with depression or ADHD require increased attention in dental care. The affected OHRQoL, especially regarding psychosocial impact, underlines the need for improved dental management of those individuals. For this purpose, an interdisciplinary care approach between psychiatric specialists and dentists should strive to foster oral health in patients with psychiatric diseases. Considering the potential response shift, the patients should be well educated, motivated, and supported regarding oral health behaviour. Future research in this field should elaborate on respective concepts and evaluate their benefit for patients.

### 4.5. Strengths and Limitations

This study systematically examined a reasonable group of patients with psychiatric diseases and, for the first time, adults with ADHD. The inclusion of a comparison group strengthens the results, too. With regard to the national reference values for OHIP G14, which were introduced by John et al. (2004), the OHIP G14 values of the comparison group were completely within the reference range for the healthy general population in Germany [39]. The applied OHIP G14 questionnaire is a validated and often used tool for research questions and appears to be a reasonable instrument for the current study. However, the oral behaviour/oral hygiene questionnaire was only composed based on previous studies but was not validated again in this study. Moreover, several limitations must be considered during the interpretation of the current study’s findings. The absence of a sample size calculation makes the statistical robustness of the sample questionable. It needs to be mentioned in this context that the group of ADHD patients was comparably small, with only 47 included patients. Therefore, it is unclear whether the statistical power is strong enough to draw meaningful conclusions. Future studies should perform a sample size calculation when studying the OHRQoL of patients with psychiatric diseases. Furthermore, the cohort of psychiatric diseases was quite heterogeneous, especially due to the inclusion of in- and outpatients. In this respect, medication for individuals with mental disease needs to be recognized. It has been reported that respective drugs, particularly antidepressants and antianxiety medication, can cause several oral side effects, mainly dry mouth, but also dysgeusia or dyskinesia [40]. Due to the complex and heterogeneous medication in the different groups in this current study, this factor was not considered but should be included in future studies to evaluate their impact on OHRQoL. Moreover, several patient-related data would be of interest for studies on OHRQoL. These include education levels, economic backgrounds, and whether they are referred by other healthcare institutions, which should be considered in future research in the field. The comparison group also has some limitations. The group originated from a patient collective from a dental clinic and visited the centre in a prevention-oriented objective. Although mental health was asked during an interview, no measures were applied to completely exclude mental disorders (e.g., screening). Additionally, similar to the psychiatrically diseased group, some more information, like economic and educational background, would be of interest to exclude potential confounders. The absence of a clinical oral examination in the cohort must also be seen as a limitation. Although the literature shows deficits in the oral health of psychiatric patients, this would require confirmation in the current study’s cohort. Future studies must include an oral examination to confirm the deficits in the oral health of psychiatric patients and provide more concrete evidence to support the current study’s findings. Although the OHIP G14 is recommendable for clinical research questions [6], recent literature suggests the usage of the OHIP 5 questionnaire [41]. Moreover, the current study is limited by its cross-sectional design. Longitudinal data would be required to evaluate the effects of oral health behaviour and respective changes on OHRQoL. Thus, future research is needed to gather longitudinal data to better understand how oral health behaviour and changes over time affect OHRQoL in patients with psychiatric diseases. Such studies also need to address potential confounders, as psychiatric patients are a special patient group.

## 5. Conclusions

Within the limitations of this current study, patients with depressive disorders and adults with ADHD show a reduced OHRQoL, which is especially pronounced in the psychosocial dimension. Additionally, a contradictory association between oral hygiene/oral health behaviour and OHRQoL was found, underlining the hypothesis of a changed perception of oral conditions by patients with mental diseases. Therefore, interdisciplinary collaboration between psychiatric specialists and dentists should be considered to foster oral health care in those individuals, whereby the patients’ perspective appears of particular relevance.

## Figures and Tables

**Table 1:** Patient characteristics in the different groups of this study.

	Psychiatric Group n = 141	Depressive Disorder n = 94	ADHD n = 47	Comparison Group N = 145	*p*-Value
Age mv ± SD	39.0 ± 13.1	40.9 ± 13.9	36.0 ± 11.3	38.9 ± 13.9	0.354
Age Range	21–67	21–67	21–65	21–67	0.653
Gender					
Male (%)	47	51	40	37	0.176
Female (%)	53	49	60	63	0.143

**Table 2:** Results of the OHIP-G14. The table shows the median values and range for the different questions and sum scores and their comparison between the study groups.

OHIP Items Mean (Range)	Psychiatric Group (n = 141)	Depressive Disorder (n = 94)	ADHD (n = 47)	Comparison Group (N = 145)	PG vs. CG *p*-Value	DD vs. CG *p*-Value	ADHD vs. CG *p*-Value	ADHD vs. DD *p*-Value
Trouble pronouncing	0 (0–4)	0 (0–4)	0 (0–2)	0 (0–2)	**0.007**	**0.003**	0.196	0.346
Taste worsened	0 (0–3)	0 (0–3)	0 (0–3)	0 (0–2)	**0.001**	0.051	**<0.001**	**0.034**
Life less satisfactory	0 (0–4)	0 (0–4)	0 (0–4)	0 (0–3)	**<0.001**	**<0.001**	**<0.001**	0.561
Difficult to relax	0 (0–4)	0 (0–4)	0.50 (0–4)	0 (0–4)	**<0.001**	**<0.001**	**0.004**	0.884
Feeling of tension	1.00 (0–4)	0.5 (0–4)	1.00 (0–4)	0 (0–4)	**<0.001**	**<0.001**	**<0.001**	0.836
Interrupting meals	0 (0–4)	0 (0–4)	0 (0–2)	0 (0–2)	**0.001**	**0.035**	**<0.001**	0.077
Uncomfortable eating	0 (0–4)	0 (0–4)	0 (0–3)	0 (0–4)	**<0.001**	**<0.001**	**<0.001**	0.197
Short tempered	0 (0–3)	0 (0–3)	0 (0–3)	0 (0–3)	**0.003**	**0.015**	**0.008**	0.555
Difficult to perform daily jobs	0 (0–4)	0 (0–4)	0 (0–3)	0 (0–4)	**0.010**	**0.017**	**0.033**	0.919
Unable to function	0 (0–3)	0 (0–3)	0 (0–2)	0 (0–3)	**0.007**	**0.026**	**0.007**	0.460
Embarrassed	0 (0–4)	0 (0–4)	0 (0–4)	0 (0–2)	**<0.001**	**<0.001**	**<0.001**	0.292
Diet unsatisfactory	0 (0–4)	0 (0–4)	0 (0–4)	0 (0–2)	**0.004**	**0.006**	**0.021**	0.994
Oral pain	1.00 (0–4)	0 (0–4)	1.00 (0–4)	0 (0–3)	**<0.001**	**<0.001**	**<0.001**	0.088
Sense of uncertainty with teeth	0 (0–4)	0 (0–4)	1.00 (0–3)	0 (0–2)	**<0.001**	**<0.001**	**<0.001**	0.396
Sum oral function	1.00 (0–16)	0 (0–16)	1.00 (0–13)	0 (0–9)	**<0.001**	**<0.001**	**<0.001**	0.090
Sum psychosocial impact	2.00 (0–25)	2.00 (0–25)	3.00 (0–19)	0 (0–16)	**<0.001**	**<0.001**	**<0.001**	0.666
Sum score	5.00 (0–46)	5.00 (0–46)	6.50 (0–38)	0 (0–31)	**<0.001**	**<0.001**	**<0.001**	0.302

Significant findings (*p* < 0.05) are highlighted in bold.

**Table 3:** Results of the questionnaire on periodontal complaints and oral health behaviour in the cohort of patients with psychiatric disease and in the two sub-groups. The absolute numbers and percentage of the respective parameters are shown.

Oral Health Behaviour and Sensation	Psychiatric Group (N = 141)	Depressive Disorder (n = 94)	ADHD (n = 47)
Gum Bleeding	54 (38.3)	35 (37.2)	19 (40.4)
Worse Taste	84 (60.4)	52 (55.9)	32 (69.6)
Periodontitis Treatment	26 (19.4)	17 (18.1)	9 (19.1)
Smoking	89 (63.6)	58 (62.4)	31 (66.0)
Regular Dental Visits	66 (47.5)	53 (57.6)	13 (27.7)
Professional Tooth Cleaning	65 (46.4)	48 (51.6)	17 (36.2)
Informed Dentist	39 (28.1)	26 (28.0)	13 (28.3)
Educated about Oral Hygiene	125 (89.3)	85 (91.4)	40 (85.1)
Interdental Cleaning	85 (60.3)	59 (62.8)	26 (55.3)
Mouth Rinse	54 (38.3)	33 (35.1)	21 (44.7)

**Table 4:** Comparison of oral health-related issues between patients with increased OHIP G14 sum score (>median, impaired OHRQoL) and low OHIP G14 sum score (not impaired OHRQoL) in patients with psychiatric disease. The absolute numbers and percentages of the respective parameters are shown.

Items	OHIP Score High (n = 67)	OHIP Score Low (n = 72)	*p*-Value
Gum Bleeding	23 (31.9)	30 (44.8)	<0.001
Smoking	44 (61.1)	43 (65.2)	<0.001
Professional Tooth Cleaning	37 (51.4)	29 (43.9)	<0.001
Educated about Oral Hygiene	66 (91.7)	58 (86.6)	<0.001
Interdental Cleaning	46 (63.9)	38 (56.7)	<0.001

## Data Availability

Data can be provided by the corresponding author upon reasonable request.

## Supplementary Materials

The following supporting information can be downloaded at: https://www.mdpi.com/article/10.3390/jcm12227192/s1. Questionnaire regarding oral hygiene and oral health behavior.
