# Supplementary material for: Oral Health-Related Quality of Life in Adult Patients with Depression or Attention Deficit Hyperactivity Disorder (ADHD)
Source: J Clin Med. 2023 Nov 20;12(22):7192. doi: 10.3390/jcm12227192 (PMC10672471; doi:10.3390/jcm12227192)
Supplement: Supplementary file 1 [file jcm-12-07192-s001.zip › jcm-2704600-supplementary.pdf]

Please answer the following questions regarding your oral hygiene, oral complaints and dental behavior.

	Yes	No
1. Do you suffer from bleading gums?	☐	☐
2. Do you perceive a bad taste/worsened taste?	☐	☐
3. Did you already undergo a periodontal treatment?	☐	☐
4. Are you smoker?	☐	☐
5. Do you visit your dentist regularly (e.g. once a year)?	☐	☐
6. Do you regularly undergo a professional tooth cleaning?	☐	☐
7. Is your dentist informed about your psychiatric disease?	☐	☐
8. Do you feel well-educated with regard to oral hygiene measures?	☐	☐

9. Do you use one of the following devices for oral hygiene?

☐ Interdental brushes or floss

☐ Mouthrinse

Thank you for participation
